# Probing the Hepatitis B Virus E-Antigen with a Nanopore Sensor Based on Collisional Events Analysis

**DOI:** 10.3390/bios12080596

**Published:** 2022-08-04

**Authors:** Ioana C. Bucataru, Isabela Dragomir, Alina Asandei, Ana-Maria Pantazica, Alina Ghionescu, Norica Branza-Nichita, Yoonkyung Park, Tudor Luchian

**Affiliations:** 1 Department of Physics, Alexandru I. Cuza University, 700506 Iasi, Romania; 2 Interdisciplinary Research Institute, Sciences Department, Alexandru I. Cuza University, 700506 Iasi, Romania; 3 Viral Glycoproteins Department, Institute of Biochemistry of the Romanian Academy, 060031 Bucharest, Romania; 4 Department of Biomedical Science and Research Center for Proteinaceous Materials (RCPM), Chosun University, Gwangju 61452, Korea

**Keywords:** nanopore, hepatitis B, antigen, monoclonal antibody, single molecule detection, electrophysiology

## Abstract

Real-time monitoring, simple operation, and cheaper methods for detecting immunological proteins hold the potential for a solid influence on proteomics and human biology, as they can promote the onset of timely diagnoses and adequate treatment protocols. In this work we present an exploratory study suggesting the applicability of resistive-pulse sensing technology in conjunction with the α-hemolysin (α-HL) protein nanopore, for the detection of the chronic hepatitis B virus (HBV) e-antigen (HBeAg). In this approach, the recognition between HBeAg and a purified monoclonal hepatitis B e antibody (Ab(HBeAg)) was detected via transient ionic current spikes generated by partial occlusions of the α-HL nanopore by protein aggregates electrophoretically driven toward the nanopore’s vestibule entrance. Despite the steric hindrance precluding antigen, antibody, or antigen–antibody complex capture inside the nanopore, their stochastic bumping with the nanopore generated clear transient blockade events. The subsequent analysis suggested the detection of protein subpopulations in solution, rendering the approach a potentially valuable label-free platform for the sensitive, submicromolar-scale screening of HBeAg targets.

## 1. Introduction

While the majority of current analytical methods rely on population-based and time-averaged information, single-molecule-based approaches reveal subpopulations of molecules as well as their interactions, allowing the description of spatial, temporal, and structural dynamic processes. In response to the pressing need for a deeper understanding of the intrinsic heterogeneity and internal dynamics of individual molecules, nanopores have emerged as highly sensitive and versatile analytical tools enabling label-free, high-throughput, and low-cost characterization of individual molecules. Nanopore sensors are extremely versatile single-molecule sensors employed for both qualitative and quantitative analysis, and representative applications include polynucleotide detection and gene sequencing [1,2,3,4,5,6,7,8,9,10,11,12,13,14], polypeptide secondary structure recognition [15,16,17,18], protein structure analysis [19,20,21,22,23], small molecule and metal ion detection [24,25,26,27,28], polymer analysis [29,30], and virus and bacteria detection [31,32,33,34,35,36,37].

The concept lying at the core of the approach originated from a patent by Wallace H. Coulter [38]. Basically, this approach involves the generation of a single nanopore (protein- or solid-state-based) on a substrate which separates volumes of the electrolyte solution, held to a transmembrane voltage difference to electrophoretically drive the analytes of interest toward the nanopore. When such analytes—with volumes similar to those of nanopore inner space—are captured at the nanopore entrance and driven through the nanopore, they displace an equivalent volume of electrolyte from the nanopore and alter its electrical resistance. Therefore, their detection is achieved through a specific blockade fingerprint, correlated with ensuing alterations in the net ionic current across it. The subsequent on- or off-line statistical analysis of the resulting current fluctuations, generates information regarding the analyte’s identity and its physical and chemical properties [39,40,41,42,43,44,45,46,47,48,49,50].

In a different set of applications, by using a combination of micropores and resistive-pulse sensing detection, the ability to perform immunoassays and detect antigen–antibody reactions was demonstrated in early 2000, by monitoring size change of latex colloids upon specific antigen–antibody binding on the colloid surface [51,52]. Later, a similar technology was employed to examine individual antibody–virus interactions [53,54]. The advent of large-scale nanopore fabrication and availability ushered in a new era in the realm of uni-molecular antibody detection and monitoring of antigen–antibody interactions [55,56,57,58,59,60,61].

As outlined above, a common prerequisite in these examples is the implication of nanopores with inner dimensions comparable to those of the antigen, antibody, or antigen–antibody complexes, so that the detection of a specific antigen–antibody binding becomes correlated with ionic-current changes caused by protein translocation through nanopores.

In this exploratory study we report the feasibility of generating a sensitive platform for detecting antigen–antibody interactions, combining the use of α-hemolysin (α-HL) nanopore-sensing technology and analysis of collisional bumping events between the nanopore entrance and target proteins, which are otherwise excluded from reversible trapping inside the nanopore. A similar strategy was previously implemented in our groups for the detection of inactivated bacteria [62].

In our proof-of-concept experiments we employed the hepatitis B virus (HBV) e-antigen (HBeAg), which is a widely used marker for both clinical management of chronic HBV infections and HBV-related basic research [63,64,65], and a purified monoclonal hepatitis B e antibody (Ab(HBeAg)). We showed that incoming proteins bumping into the nanopore’s vestibule entrance determine stochastic ionic current changes through an α-HL nanopore and enable their detection, even in the absence of excluded volume measurements.

## 2. Materials and Methods

### 2.1. Reagents

The purified HBeAg used herein was produced in *E. coli,* as described below. The antigen was fused with a six-histidine-residues tag at the C-terminus, to enable easy (one-step) purification of sufficient amounts for further analysis. Alternatively, a commercially available antigen denoted herein by HBeAg* expressed without tags (Mw = 17, kDa, pI = 6.98), was purchased from Origene, Rockville, Maryland, USA (# BIN049) and used as control. Given that in addition to the commercial HBeAg*, the purified HBeAg contained six histidine residues (pI = 7.64 at 25 °C), its net electric charge at pH = 8 as used herein, remained anionic. The anti-HBeAg antibody, Ab(HBeAg) (Mw = 155 kDa, pI ~ 7.0) was purchased from Santa Cruz Biotechnology, Dallas, Texas, USA (#sc-51936).

Potassium chloride (KCl), human serum (HS), n-pentane, hexadecane, 4-(2-hydroxyethyl)-1-piperazineethanesulfonic acid (HEPES), potassium hydroxide, and α-hemolysin (α-HL) were purchased from Sigma-Aldrich (Darmstadt, Germany). The 1,2-diphytanoyl-sn glycerophosphocholine (DPhPC) lipids were supplied by Avanti Polar Lipids (Alabaster, AL, USA).

### 2.2. Protein Preparation

#### 2.2.1. HBeAg Cloning

The DNA sequence encoding for the mature HBeAg was first amplified by PCR using the pTriExHBV1.1 template, which contains 1.1 units of the entire HBV genome [66] and the following primers: Fw: 5′- CAT ATG TCC AAG CTG TGC C -3′; Rev: 5′- GCTC GAG AAC AAC AGT AGT C -3′. The PCR fragment was further cloned into the pET-24a, in frame with six His residues at the C-terminus, to produce the pET-24a-HBeAg plasmid. Insertion of the HBeAg coding region was verified via agarose gel electrophoresis and confirmed by DNA sequencing.

#### 2.2.2. HBeAg Expression

The pET-24a-HBeAg plasmid was used to transform *E. coli* DE3-RIL cells, which contained supplementary arginine, isoleucine and leucine codons. A single colony was then inoculated in 100 mL of Luria-Bertani (LB) medium supplemented with kanamycin (50 µg/mL) and incubated overnight at 37 °C and shaking at 130 rpm. The bacterial culture was diluted 1:20 in 1 L of LB medium supplemented with kanamycin (50 µg/mL) and incubated at 37 °C and shaking at 130 rpm until the optical density of the culture reached 0.8. Protein expression was induced by adding 1 µM IPTG to the cells and culturing for another 4 h. The cells were then collected via centrifugation at 15,000× *g* for 20 min and further processed for purification.

#### 2.2.3. HBeAg Purification

The bacterial cells expressing HBeAg were lysed in Ni–NTA binding buffer (20 mM imidazole, 500 mM NaCl, 20 mM HEPES, pH = 8) supplemented with 10% Triton-X, 0.1% PMSF, lysozyme (0.1 mg/mL), 2 µM DTT, and protease inhibitor cocktail (1X), by sonication. The lysate was clarified via centrifugation at 15,000× *g* for 20 min and the supernatant was retained for further purification using a HisTrap HP column (1 mL, Cytiva). The column was first washed with 5 column-volumes (CV) of distilled water, followed by 10 CV of Ni–NTA binding buffer. The sample was loaded onto the column at a flow rate of 0.2 mL/min to ensure proper binding. The column was then washed with 20 CV of binding buffer prior to elution to remove contaminants. The elution step was performed using a gradient of binding buffer and elution buffer (500 mM imidazole, 500 mM NaCl, and 20 mM HEPES, pH = 8) and collected fractions were then analyzed via SDS-PAGE and Coomassie blue staining. To determine whether the obtained antigen was in monomeric or dimeric form the analysis was performed under both native and reduced (+DTT) conditions. This analysis revealed that the HBeAg was predominantly produced as a monomer. Recognition of the obtained antigen by anti-HBe antibodies was further demonstrated by using a commercial Monolisa HBe Ag–Ab PLUS (Bio-Rad). Typically, our protocol for production and one-step purification of the HBeAg leads to high protein yields (1,2 mg protein/mL) and a suitable purity for subsequent analysis (~80%).

#### 2.2.4. HBeAg Quantification

The antigen yield was quantified using an in-house ELISA. Briefly, 96-well plates (Costar) were coated using serial dilutions (500–0.015 ng/well) of the commercial HBeAg, overnight at 4 °C. Afterwards, the plates were washed three times with PBS supplemented with 0.1% Tween-20 (PBS-T) and blocked in 10% skim milk for 1 h at room temperature (RT) followed by washing three times with PBS-T. The plates were then incubated with anti-HBeAg antibodies (1:1000) for 1 h at RT and washed five times with PBS-T, followed by incubation with HRP-conjugated mouse IgGk light-chain-binding protein (#sc-516102, Santa Cruz Biotechnology, 1:10,000) for 1 h at RT. Then, the plates were washed five times with PBS-T and incubated with TMB for 30 min and the reaction was stopped by the addition of 2N H_2_SO_4_. Protein detection was performed by reading the optical density at 450 nm. The HBeAg stock solutions were split into aliquots and stored at −20 °C. Prior to particular experiments, the samples were brought to room temperature.

### 2.3. Single-Molecule Electrophysiology

Measurements were carried out as previously reported [67]. The recording cell consisted of two chambers of 1 mL volume termed *cis* (grounded) and *trans*, separated by a 25 μm-thick Teflon film (Goodfellow, Malvern, MA, USA) containing an aperture of ~120 μm in diameter. Following the pre-treatment of the aperture with a 1:10 hexadecane/pentane (HPLC-grade) solution, planar lipid membranes made of 1,2-DPhPC lipids were obtained using the Montal–Mueller approach [68]. Equal amounts of a slightly basic (pH ~8) electrolyte solution made of 2 M KCl buffered with 10 mM HEPES were added on both chambers of the bilayer cell. Following *cis*-side addition of small amounts from a monomeric α-HL protein stock solution prepared in 0.5 M KCl, gentle stirring led to the formation in the lipid bilayer of a single homo-heptameric α-HL nanopore. Next, HBeAg or HBeAg*, and the Ab(HBeAg)—present alone or pre-incubated for 2 h 30 m at room temperature with the antigen—were pipetted at particular concentrations to the *cis* side of the membrane accessible to the vestibule entrance of the nanopore and subjected to a transmembrane potential. Ionic current fluctuations generated by protein interactions with the α-HL’s vestibule were recorded in voltage-clamp mode via two Ag/AgCl electrodes connected to an Axopatch 200B amplifier (Molecular Devices, San Jose, CA, USA) and low-pass filtered at 10 kHz. The signals were fed into a NI PCI 6221, 16-bit acquisition board (National Instruments, Austin, TX, USA), operating at a sampling frequency of 50 kHz within LabVIEW 8.20 (National Instruments, USA), to enable the digital recording of electrical signals. The bilayer chamber was shielded in a Faraday cage (Warner Instruments, Hamden, CT, USA) and placed on a vibration-free platform (BenchMate 2210, Warner Instruments, USA) to limit the influence of external noise. The numerical analysis and graphic representations of the data collected were undertaken with pClamp 6.03 (Axon Instruments, San Diego, CA, USA) and Origin 6 (Origin Lab, Northampton, MA, USA) software.

### 2.4. Representation of Molecular Structures

The graphic representations of the HBeAg were produced with the molecular visualization software RasTop 2.1 (http://www.geneinfinity.org/rastop/ accessed on 13 July 2022) by retrieving the Protein Data Bank (PDB) atomic coordinates that described the crystal structure of the HBeAg antigen (PDB ID: 3V6Z) [69,70]. The monomeric structure of the HBeAg was selected and physical dimensions were inferred. The anti-HBeAg antibody and α-HL protein nanopore molecular structures were represented using the web app for 3D visualization Mol* Viewer [71] available on RCSB PDB (https://www.rcsb.org/ accessed on 13 July 2022) [72], using the crystallographic structure of immunoglobulin (PDB ID: 1IGY) [73,74], and the structure of staphylococcal α-HL (PDB ID: 7AHL) [75,76].

## 3. Results and Discussion

- *Experimental Principle*

**Figure 1:**
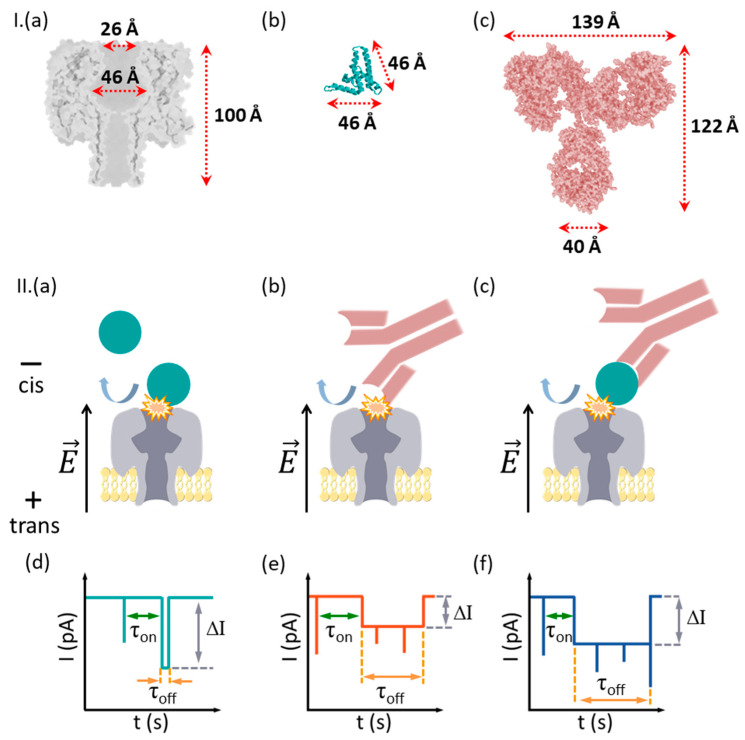
Simplified description of the measurement principle. (**I**) In (**a**–**c**) are presented at a similar scale the homo-heptameric α-HL protein nanopore, HBeAg protein antigen and its monoclonal antibody Ab(HBeAg). (**II**) Due to the exclusion volume effects, the nanopore cannot capture and accommodate inside it the HBeAg (**a**), Ab(HBeAg) antibody (**b**), or HBeAg–Ab(HBeAg) complexes formed in solution (**c**). The electrophoresis of anionic HBeAg, Ab(HBeAg) antibody, or HBeAg–Ab(HBeAg) complexes toward the nanopore result in collisional interactions with the nanopore, seen as stochastic spikes in the ionic current (**d**–**f**), correlated with the transient obturation of the α-HL’s vestibule entrance area by an incoming analyte. Such events are characterized by the blockade depth (ΔI), dwell time (τ_off_), and inter-events intervals (τ_on_). In certain cases, a series of fast-occurring spikes are seen during a single collisional event (see schematics in panels e and f, the capture events), which we posit to reflect stochastic, spatial re-arrangements of the captured analyte at the nanopore’s mouth.

As reported above, the detection setup comprised a reconstituted lipid membrane with an isolated single α-HL nanopore, separating electrolyte solutions into two compartments conventionally named *cis* (grounded) and *trans*. When *cis*-side-added analytes collide with the α-HL’s vestibule entrance, ionic current blockades ensued and such stochastically repetitive events were recorded as a continuous alteration of the current signal (Figure 1).

In a critical departure from previously implemented paradigms involving detection with nanopores (vide supra), and due to the larger volume of the protein analytes as compared to α-HL inner compartments, precluding their capture inside the nanopore, the challenge herein was to probe the utility of the techniques when the available signals arose solely from analyte–nanopore collisional, bumping events.

- *HBeAg and Ab(HBeAg) proteins interact with the α-HL’s vestibule entrance and generate collisional-based alterations of the ionic current*

In a first series of experiments, we studied the interaction of the α-HL nanopore with either the HBeAg antigen protein or its purified monoclonal antibody (Ab(HBeAg)), added alone on the bilayer-membrane cell. Due to its pI (vide supra), the monomeric HBeAg antigen protein is negatively charged above neutral pH, so that *trans*-positive electric potentials were expected to drive electrophoretically the HBeAg toward the nanopore vestibule entrance (Figure 1II).

**Figure 2:**
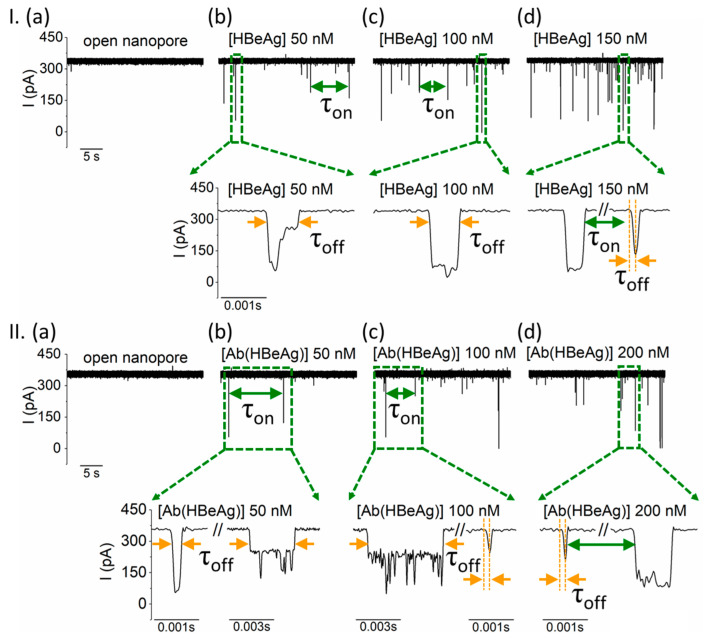
Despite their relative large size, the interactions between the *cis*-side-added HBeAg and Ab(HBeAg) with the α-HL nanopore are visible in electrophysiology experiments. (**I**) Reversible collisional interactions between the positively biased α-HL (**a**) and the *cis*-side-added HBeAg, present at 50 nM (**b**), 100 nM (**c**), and 150 nM (**d**) are seen as downwardly oriented spikes. The expanded traces illustrate the degree of heterogeneity of the blockade events, suggestive of geometrical re-orientations or/and structural changes of the captured analyte during individual bumping events. (**II**) The ionic current through a positively biased nanopore (**a**) is transiently blocked when the monoclonal antibody Ab(HBeAg) is added on the *cis* side of the chamber at incremental concentrations of 50 nM (**b**), 100 nM (**c**), and 200 nM (**d**), as a result of transient occlusions of the vestibule entrance of the nanopore during collisions. As presented in the expanded traces, the rich diversity of blockade substates manifested during individual collisional events, all characterized by various τ_off_ values, supports the hypothesis of a highly dynamic state of the transiently captured antibody in terms of orientation and/or tertiary structure, all leading to various degrees of nanopore occlusion. The traces were recorded at an applied potential ΔV = +180mV in a 2 M KCl electrolyte solution, buffered at pH = 8 with a 10 mM HEPES solution.

As shown in Figure 2I(a), in a buffer containing 2 M KCl, 10 mM HEPES, pH = 8, the ionic current through an open α-HL nanopore held at +180 mV equaled 339.87 ± 9.52 pA. Subsequent and successive *cis*-side additions from the purified HBeAg antigen protein generated stochastic reductions in the ionic through the nanopore (Figure 2I(b–d)). As evidenced in Figure 1, given the larger size of HBeAg with respect to the nanopore vestibule entrance of ~2.6 nm in diameter, the blocking events reported in Figure 2I(b–d) reflect electrophoretically-assisted, collisional bumping events of individual HBeAg proteins with the nanopore, followed by a physical blocking of it and release on the same side. As a positive control for the production of the synthesized HBeAg antigen protein, similar experiments were also carried out with the commercially available HBeAg*, which confirmed the results obtained with our purified HBeAg protein (Figures S1 and S2).

It should be noted that previous research demonstrated that natively, HBeAg is a disulfide-bond-linked dimer protein [69,77]. In line with this knowledge, in a preliminary control experiment we noticed that ionic current fluctuations associated with the transient capture of repetitively ‘froze-thawed’ HBeAg at the vestibule entry of the positively biased nanopore, decreased visibly. Most likely, this reflected the formation of dimeric, oxidized HBeAg, whose conformational and physical features diminished its capture propensity at the α-HL’s vestibule entrance (Figure S3). To minimize such oxidative processes in all subsequent experiments, the antigen protein was partitioned in distinct aliquots which were thawed and used only once during electrophysiology recording.

In another set of experiments, we observed that *cis*-side addition of Ab(HBeAg) at various aqueous concentrations, led to similar patterns of ionic current blockades through the positively-biased nanopore, also reflecting bumping events with the nanopore’s vestibule entrance (Figure 2II). As expected, these interactions appeared to be absent at negative potentials (Figure S4), due to the anionic charge of the monoclonal antibody Ab(HBeAg) at pH = 8.

**Figure 3:**
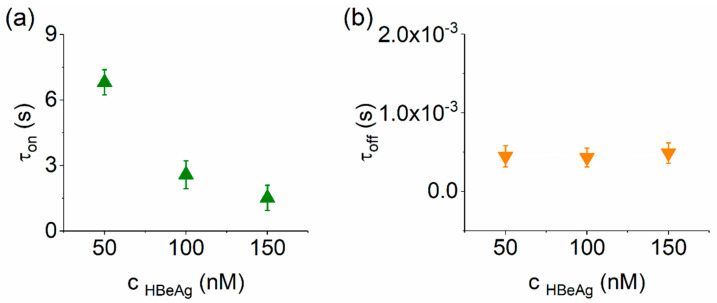
Quantitative analysis of HbeAg–α-HL reversible interactions. (**a**) Concentration dependence of the time intervals reflecting the purified HbeAg-α-HL association (τ_on_; **a**) and, respectively, dissociation (τ_off_; **b**), measured at an applied potential ΔV = +180 mV.

To further assess our technique’s ability to successfully detect HBeAg proteins, we statistically analyzed such interactions with the nanopore by means of the average blockade duration (τ_off_) and that measured between consecutive blockade events (τ_on_) (Figure 3). To this end, complications arose from two main sources: (i) Unlike situations whereby smaller analytes are captured inside the nanopore and translocation, thus generating reproducible events in terms of relative current blockade. The events statistics in the present case are complicated due to the emergence of multiple classes of current blockade signals (Figure 2). As suggested, the broadening of the signal distributions in terms of blockade amplitudes most likely reflects orientation effects related to the relative position of the incoming proteins to the nanopore vestibule entrance during temporary binding events. (ii) Rapid blockades, comparable to the rise time of the low-pass filter used in the experiments ($\tau_{rise}\approx \frac{0.33}{f_{corner}}=33\;\mu s$, where $f_{corner}$ = 10 kHz) were prone to distortion by the limited bandwidth (0, $f_{corner}$) of the amplifier.

To alleviate the first challenge and depending on the experiment, the average blockade duration of blockade events was calculated as shown in Figure 2Ib or Figure 2IIb,c, which is ignoring the intermediate ionic current flickering visible during a single collisional event. Further, to accurately recover the width of short ionic current pulses, we used a protocol referenced previously [78]. Briefly, the duration measured between the start of the blockade event and the data point corresponding to local minimum of the ionic current measured during that event was assigned to the event duration (τ_off_) (see expanded traces in Figure 2I(d) and Figure 2II(c,d), signals in orange).

With these considerations, the analysis presented in Figure 3 reveals that the average time for HBeAg capture by the nanopore decreased with the increase in the analyte concentrations, whereas the dissociation time remained constant. The main take-away from such experiments is that the electrodiffusion-mediated collision of the HBeAg with the α-HL nanopore vestibule entrance is consistent with a bimolecular-like reaction scheme. In support of this hypothesis, we demonstrated in a complementary analysis that the average capture time of the anionic Ab(HBeAg) protein by the nanopore decreased with the increase in the analyte concentrations and the applied positive potential (Figure S5).

Altogether, these data establish that nonspecific HBeAg–nanopore interactions on the outside region around the nanopore’s vestibule entrance are still capable of modulating the ionic current across the nanopore, lending support for the ability of this approach to sense such relatively large individual proteins.

- *Nanopore detection of HBeAg–Ab(HBeAg) complexes*

Having established that aqueous HBeAg and Ab(HBeAg) proteins associate reversibly via simple collisions with the α-HL vestibule entrance, seen in a reproducible manner via blockades of the ionic current through the nanopore, we then examined the possibility of detecting the specific formation of antigen–antibody complexes. As depicted in Figure 1, the underlying rationale is simple: as antigen–antibody formation entails conformational, volume distribution and electrical charge changes on the complex relative to either antibody or antigen proteins alone, we posit that antigen–antibody complexes would be visible through alterations in the nanopore-mediated ionic current blockades pattern and therefore provide a test case for nanopore-based HBeAg–Ab(HBeAg) interaction studies.

**Figure 4:**
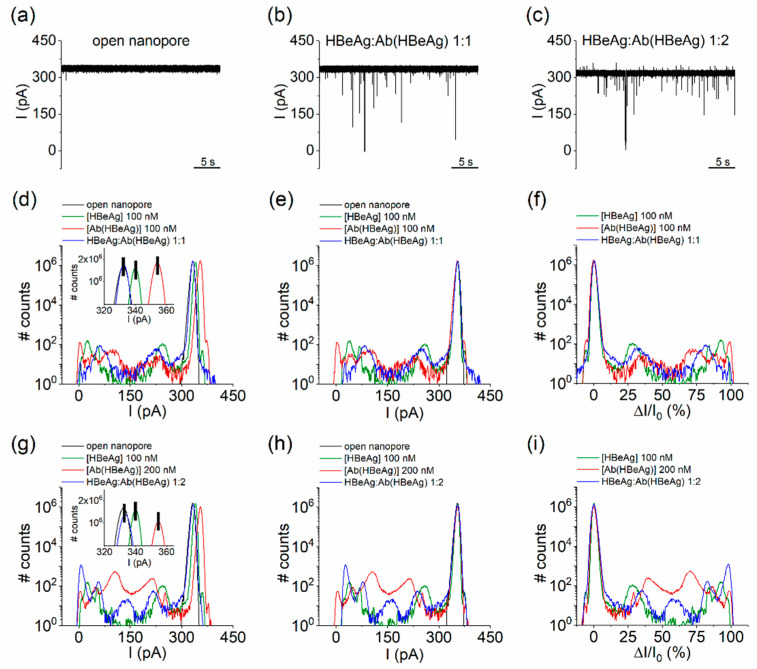
Detection of HBeAg–Ab(HBeAg) complexes with the α-HL nanopore. (**a**–**c**) Interaction between α-HL and nonincubated, *cis*-side-added HBeAg and Ab(HBeAg) mixed at a 1:1 (100 nM:100 nM) and 1:2 (100 nM:200 nM) molar ratio generate solid ionic currents blockades across the open α-HL nanopore at an applied voltage ΔV = + 180 mV. (**d**) All-points histograms showing the distinct conductive states of α-HL nanopore, while interacting with the *cis*-side-added HbeAg, Ab(HBeAg), or HBeAg–Ab(HBeAg) complexes (1:1 molar ratio). In the inset we represent a zoomed-in excerpt evidencing the experimental shift in the open pore current through the nanopore in either case (see also text). (**e**) Shift-corrected histograms, all aligned to a similar value corresponding to the open nanopore current. (**f**) The shift-corrected, all-points histograms transformed in terms of percent relative current blockades (% $\frac{∆I}{I_{o}}$, where ∆I is the current magnitude of a blockade substate relative to the open nanopore current $I_{o}$). (**g**) All-points histograms showing the distinct conductive states of α-HL nanopore, while interacting with the *cis*-side-added HbeAg, Ab(HBeAg), or HbeAg–Ab(HBeAg) complexes (1:2 molar ratio). As above, in the inset we represent an excerpt displaying the experimental variability in the open pore current through the nanopore during such measurements. (**h**) Shift-corrected histograms, all aligned to a similar value corresponding to the open nanopore current. (**i**) The shift-corrected, all-points histograms transformed in terms of percent relative current blockades (% $\frac{∆I}{I_{o}}$).

Starting from experiments carried out as displayed in Figure 2, in which the interaction of the *cis*-side-added purified HBeAg (100 nM) with the nanopore was evidenced, subsequent incremental addition of the Ab(HBeAg) to achieve concentrations of 100 and 200 nM, respectively, in a recording solution, led to the appearance of new blockage peaks on the recorded signal (Figure 4d,g, blue lines), distinct from the individual blockage distribution recorded when either the Ag(HBe) or the Ab(HBeAg) was present alone in the buffer (Figure 2). To eliminate the contribution of statistical variability of the ionic current through the open nanopore to the effects seen with the reagents added (readily visible in the insets of Figure 4d,g), all points histograms were manually shifted so as to precisely superimpose the peaks corresponding to open pore current distributions. This allowed us to compare the position of the relative current blockade peak maxima ($\frac{∆I}{I_{o}}$), and accurately pinpoint the essential differences in the ensuing conductance fluctuations of the nanopore in the absence of (open pore), and, respectively, successive additions of the HBeAg, Ab(HBeAg), or HBeAg–Ab(HBeAg) complexes.

Noticeably, the formation of HBeAg–Ab(HBeAg) complexes at 1:2 molar ratio led to the emergence of a new blockade substate centered around 158.57 ± 0.6 pA (Figure 4h,i, blue curves), accompanied by the apparent cessation of the substates suggestive of Ab(HBeAg)–nanopore interactions, centered around 105.09 ± 0.12 pA and 210 ± 0.43 pA (Figure 4h,i, red curves). At the same time, the blockade substates suggestive of HBeAg–nanopore interactions were preserved, despite of a small shift in amplitude as compared to the case when the antigen protein was added alone (Figure 4h,i, green curves).

To further interpret this finding toward the identification of HBeAg–Ab(HBeAg) complexes from such a blockade signature, one must also tackle their binding stoichiometry. In a previous study using the recombinant e-antigen (rHBeAg) in homodimer form and a panel of chimeric rabbit/human monoclonal antibody fragments (Fab), a 2:2 and 1:2 (rHBeAg:Fab) binding stoichiometry was observed, indicating that one Fab binds to a rHBeAg monomer or alternatively, the binding of one antibody to a dimer blocks either sterically or allosterically binding of a second antibody [63]. Based on the above results, we hypothesized that, most likely, the newly appeared current blockades presented in Figure 4h,i were caused by individual HBeAg–Ab(HBeAg) molecular species presenting a 1:1 binding stoichiometry, bumping into the nanopore. However, we cannot rule out that the buffer may also contain post-purification products, such as structural isomers or aggregated proteins.

When similar experiments were carried out with the HBeAg–Ab(HBeAg) mixture (1:2) pre-incubated for 2h 30min at room temperature, a largely similar result was seen, in the sense that HBeAg–Ab(HBeAg)–nanopore interactions generated a new, yet less prevalent, blockade substate around 169.43 ± 1.23 pA, while the substates accompanying Ab(HBeAg)–nanopore interactions, centered around 105.09 ± 0.12 pA and 210 ± 0.43 pA were considerably reduced (Figure S6). Therefore, we concluded that nanopore detection of HBeAg–Ab(HBeAg) complexes could be obtained in real time, during successive addition of the antigen and antibody proteins, without the need for a preceding incubation period.

- *Noise analysis of ionic current fluctuations generated by HBeAg, Ab(HBeAg), or HBeAg–Ab(HBeAg) complex interactions with the α-HL*

While the detection of monomeric HBeAg, Ab(HBeAg), or HBeAg–Ab(HBeAg) complexes was possible from recordings reflecting collisional analyte–nanopore events, a strong drawback regarding the possibility of accurately inferring physical properties (e.g., volume, electric charge) of such proteins with the nanopore arises because the ~ 2.6 nm in diameter nanopore vestibule entrance is smaller than analyte sizes. However, alterations in the overall kinetics of the molecular bumping events seen while the studied proteins collide with a single α-HL nanopore, quantified via spectral analysis of ensuing ionic current fluctuations, have the potential to provide an efficient instrument for detecting HBeAg–Ab(HBeAg) formation.

As known, aside from the analyte-induced ionic current noise across the nanopore, other contributing sources prevail across distinct frequency bandwidths. For instance, at low frequencies, the noise spectrum is dominated by the flicker noise (1/f noise) stemming from the nanopore itself, whereas dielectric noise arises in the high frequency regime [79,80]. To alleviate such low-frequency noise contributions, the spectral analysis of ionic current fluctuations was performed in the range from 25 to 3000 Hz.

**Figure 5:**
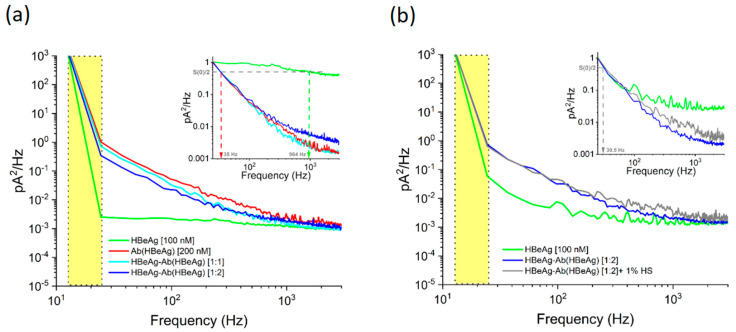
The noise analysis of collisional events ensued by proteins interacting with the α-HL nanopore (**a**) Representative power-spectra of the ionic current fluctuations recorded at ΔV = +180 mV, associated with the HbeAg, Ab(HBeAg), or HbeAg–Ab(HBeAg) complex collisions with a single α-HL nanopore. (**b**) The power spectrum of current fluctuations induced by HbeAg–Ab(HBeAg) complexes interacting with the α-HL nanopore remained unaltered in the presence of 1% human serum (HS). The yellow domains represent the frequency bandwidth containing excessive low-frequency noise contributions, subsequently excluded from the analysis (see also main text).

The analysis of the power spectral density for current fluctuations associated with HBeAg, Ab(HBeAg), and HBeAg–Ab(HBeAg) complex interactions in the α-HL nanopore revealed that such analytes generated distinct amplitudes in the low-frequency domain (Figure 5a). As seen, the power spectral density amplitudes of fluctuations caused by the HBeAg–Ab(HBeAg) complex interactions with the nanopore, were visibly reduced as compared to those mediated by Ab(HBeAg) alone, and the ionic current fluctuations displayed enhanced amplitudes as compared to those induced by the HBeAg. Moreover, the characteristic frequency (f(Hz) at $\frac{S\left(0\right)}{2}$) corresponding to the half-reduction of the maximum power spectrum density—measured herein at 25 Hz (S(0))—presented the largest value in the presence of HBeAg alone, and exhibited a considerable decrease when either Ab(HBeAg) or HBeAg–Ab(HBeAg)–α-HL interactions were studied (Figure 5a, the normalized inset).

Thus, the noise spectral density analysis contributes key information for distinguishing such proteins; however, solely by itself, noise analysis is insufficient for accurate analyte identification. For example, the characteristic frequencies for Ab(HBeAg) or HBeAg–Ab(HBeAg)–α-HL interactions were largely similar (Figure 5a, the normalized inset), the sole distinction being noticed with regard to the absolute amplitudes of the power spectral density for current fluctuations.

Despite such shortcomings, we envision that by capitalizing on the implementation of machine-learning-based classification algorithms [37,81,82] quantifying event durations and areas and parameters stemming from fluctuations analysis, the presented system could serve as an efficient, simple-to-use, and unexpensive model for subpopulation detection in complex protein mixtures.

**Figure 6:**
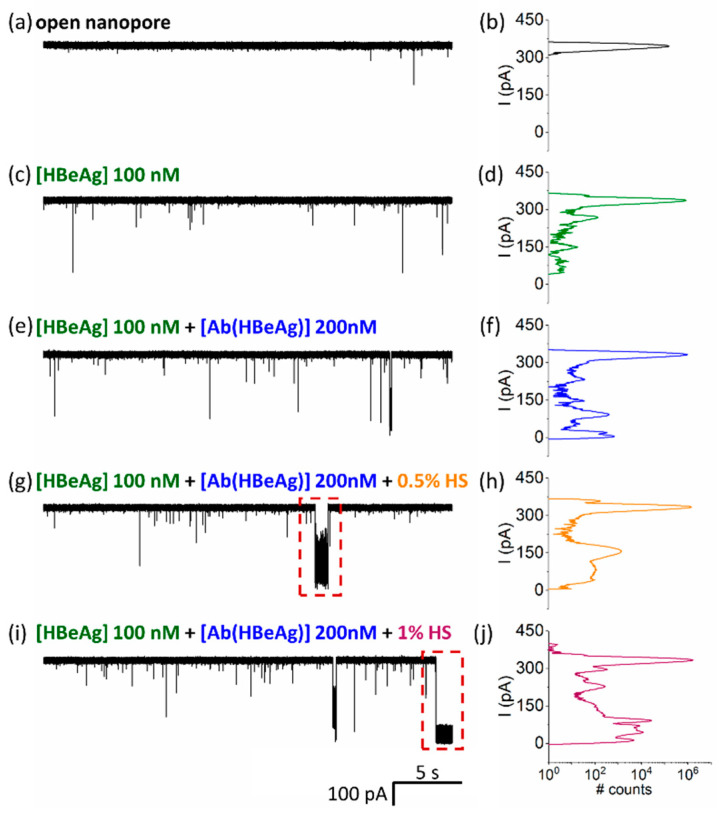
HbeAg–Ab(HBeAg) complex detections with a single α-HL nanopore in the presence of human serum proteins. Representative, original traces and related all-points histograms of the ionic current measured at ΔV = +180 mV across an open nanopore (**a**,**b**), then in the presence of *cis*-added Ag(HBe) antigen (100 nM) (**c**,**d**), followed by addition of the Ab(HBe) antibody (200 nM) (**e**,**f**), and subsequent pipetting of human serum (HS) at a 0.5% (*v*/*v*) (**g**,**h**) and, respectively, 1% (*v*/*v*) concentration (**i**,**j**). In certain cases, the HS addition led to the non-specific occlusion of the nanopore (red rectangle in panels (**g**,**i**), followed in certain cases by lipid membrane rupture.

Given the fact that in practical applications of antigen detection, a sample will contain other serum proteins, we next investigated the effects of a heterogeneous protein sample on the detection of antigen as implemented above, to further underline the possible utility of the proposed sensing paradigm in clinical-disease diagnosis. As revealed in Figure 6, incremental addition of human serum (HS) to achieve dilutions of 0.5% and, respectively, 1% in the nanopore cuvette, led to the broadening of peaks in the all-point histogram associated with the blockade events caused by HBeAg–Ab(HBeAg) conjugated complex– nanopore interactions, and the appearance of new blockage peaks consistent with the interaction of nonspecific macromolecules in human serum interactions with the nanopore. An important finding was that Ag(HBe):Ab(HBe) conjugated complexes, as evidenced by the blockade substate centered around 147.82 ± 0.32 pA, remained visible even in serum solution. Therefore, such events can be easily filtered out using a machine-learning algorithm, enabling specific detection and discrimination of antigen:antibody binding from background events that are present due to serum proteins.

To additionally support this analysis, the power spectral density calculated for current fluctuations associated with HBeAg–Ab(HBeAg) complex interactions in the α-HL nanopore, revealed that HBeAg–Ab(HBeAg) complexes elicited ionic current fluctuations whose amplitudes distribution remained practically unaltered following the human serum addition in the sample (Figure 5b). However, we admit that beyond this result, an exhaustive selectivity testing of the presented single-molecule approach toward other proteins encountered in real-life matrixes is required, before deploying such an analytical method in real-life investigations.

## 4. Conclusions

By employing the α-HL-based nanopore sensing technology, we propose a label-free, simple and sensitive assay for HBeAg–Ab(HBeAg) complex detections in a complex mixture at nM concentration, despite the relatively large volume of all analytes that prohibit their capture inside the nanopore. All-points histogram and spectral analysis of ionic current fluctuations measured across a single α-HL nanopore, reveal that sequential *cis*-side addition and mixing of nM concentrations of HBeAg and a monoclonal antibody, Ab(HBeAg), generates blockade signals distinct from those measured in the presence of either analyte alone, which presumably reflect the antibody-antigen complex formation. The signature of the HBeAg–Ab(HBeAg) blockade signal is un-altered by antigen and antibody pre-incubation.

While admitting the conceptual challenge associated with the fact that unlike other applications, the α-HL nanopore cannot host inside the herein studied proteins to ensure more accurate blockade signatures, our findings suggest that unlabeled nanopore detection can still be used to qualitatively detect HBeAg and their antibody-conjugated complexes. As the nanopore sensing approach suffers from limitations intrinsically linked to employing single-molecule detectors, e.g., meaningful extraction of parameters from sequential uni-molecular analyte-nanopore interactions resulting in a heterogenous collection of blockade events, machine learning approaches would greatly simplify the identification of specific antigen–antibody interaction’s signatures and provide a new method for antigen detection or drug screening design.

## Data Availability

Not applicable.

## Supplementary Materials

The following are available online at https://www.mdpi.com/article/10.3390/bios12080596/s1, Figure S1: Bumping interactions between the α-HL nanopore and HBeAg* are visible through electrophysiology recordings; Figure S2: Statistical analysis of α-HL-HBeAg* interactions; Figure S3: HBeAg monomers dimerize in aqueous solution and are precluded from the interaction with the α-HL vestibule entrance; Figure S4: The polarity of the transmembrane voltage critically sets the occurrence of monoclonal Ab(HBeAg)-α-HL nanopore interactions; Figure S5: The concentration- and voltage-dependent association of the monoclonal Ab(HBeAg) antibody with a single α-HL nanopore; Figure S6: Detection of pre-incubated HBeAg-Ab(HBeAg) complexes with the α-HL nanopore.
