# Supplementary material for: Probing the Hepatitis B Virus E-Antigen with a Nanopore Sensor Based on Collisional Events Analysis
Source: Biosensors (Basel). 2022 Aug 4;12(8):596. doi: 10.3390/bios12080596 (PMC9405897; doi:10.3390/bios12080596)
Supplement: Supplementary file 1 [file biosensors-12-00596-s001.zip › biosensors-1841315-supplementary.pdf]

Supplementary Information for:

Probing the Hepatitis B virus e-antigen with a nanopore sensor based on collisional events analysis

Ioana C. Bucataru[1,#], Isabela Dragomir[2,#], Alina Asandei[2], Ana-Maria Pantazica[3], Alina Ghionescu[3], Norica Branza-Nichita[3], Yoonkyung Park[4,*], Tudor Luchian[1,*]

[1]Department of Physics, Alexandru I. Cuza University, 700506 Iasi, Romania

[2]Interdisciplinary Research Institute, Sciences Department, Alexandru I. Cuza University, 700506 Iasi, Romania

[3]Viral Glycoproteins Department, Institute of Biochemistry of the Romanian Academy, 060031 Bucharest, Romania

[4] Department of Biomedical Science and Research Center for Proteinaceous Materials (RCPM), Chosun University, Gwangju, Republic of Korea, 61452

#These authors contributed equally

*Corresponding authors: luchian@uaic.ro (T.L.); y_k_park@chosun.ac.kr (Y.P.)

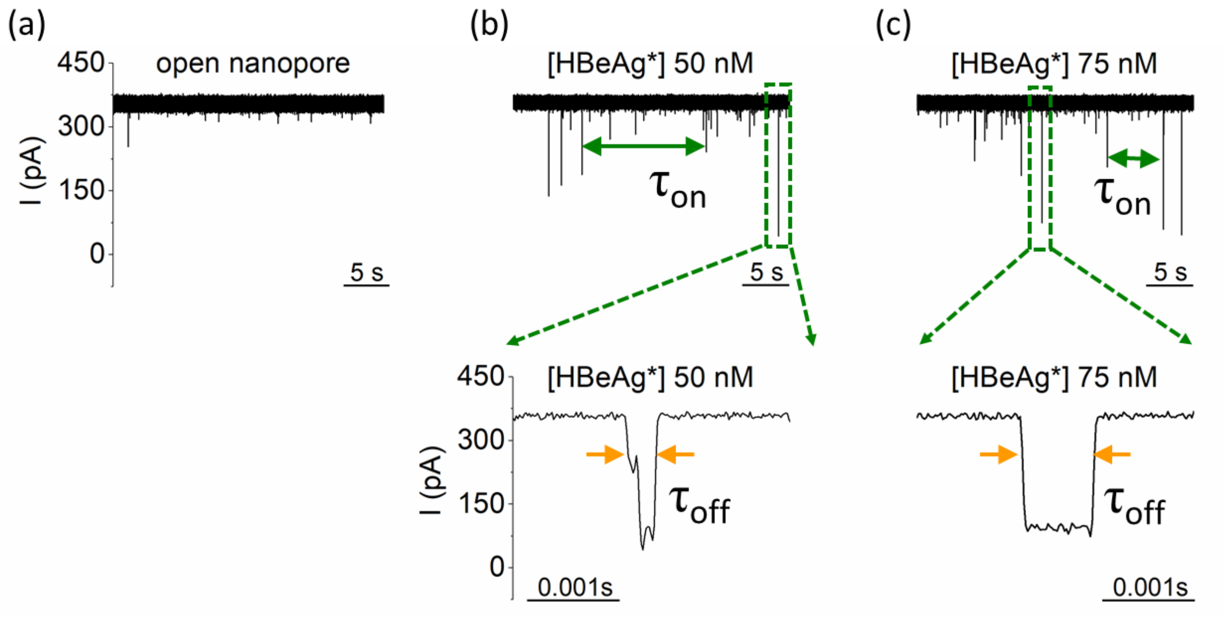

Figure S1. **Bumping interactions between the α-HL nanopore and HBeAg* are visible through electrophysiology recordings.** The ionic current through the α-HL (a) gets reversibly blocked (downwardly oriented spikes) upon nanopore interactions with the *cis*-side added HBeAg* at various concentrations of 50 nM (b) and respectively 75 nM (c). In the expanded traces we display characteristic HBeAg*-induced blockade events. The traces were recorded at an applied potential ΔV = +180mV in a 2M KCl electrolyte solution, buffered at pH = 8 with a 10 mM HEPES.

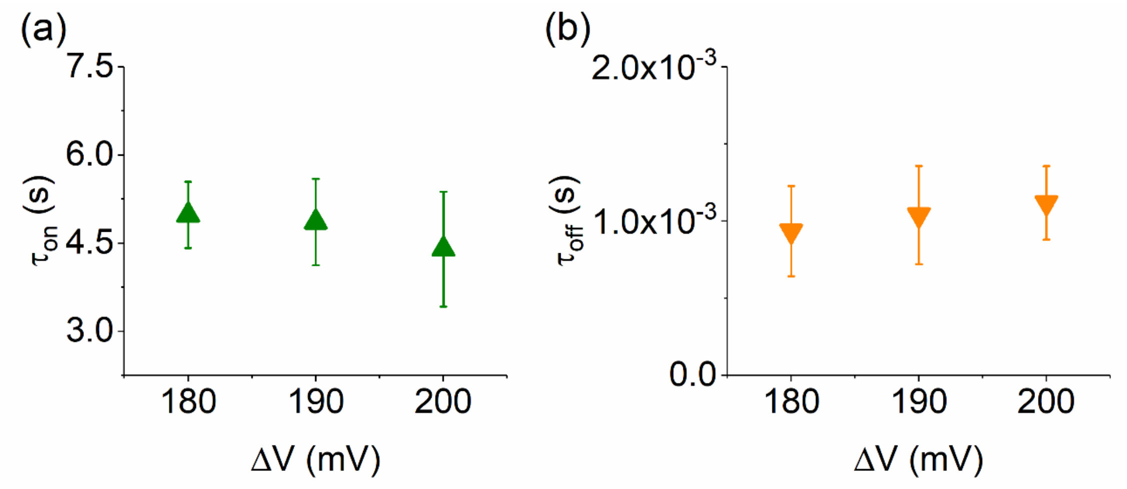

Figure S2. **Statistical analysis of α-HL-HBeAg* interactions.** (a) In accord with the expected physical scenario, the negatively charged, *cis* side-added HBeAg* protein interacts more favorably with a single α-HL nanopore at increasing positive values of the holding potential, as suggested by the small, yet consistent decrease of the inter-events average time intervals (τ_{on}). (b) The average dissociation time intervals (τ_{off}) slightly increase with the increase in the holding potential across the positively biased nanopore, suggesting the release of the HBeAg* in the same chamber upon unbinding from the nanopore.

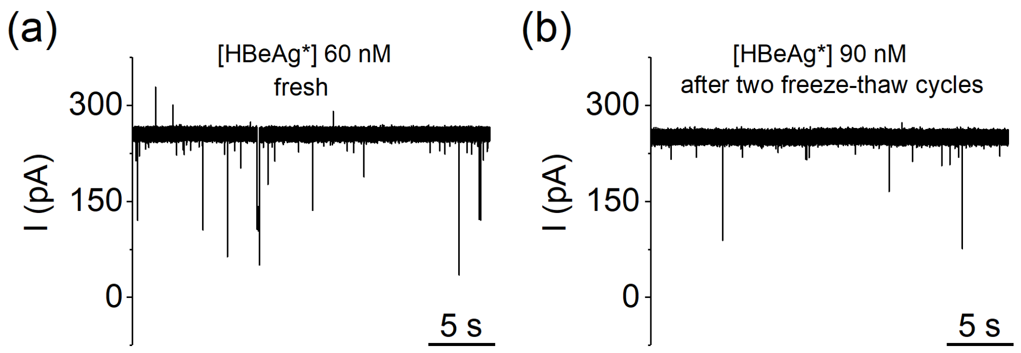

Figure S3. **HBeAg monomers dimerize in aqueous solution and are precluded from the interaction with the α-HL vestibule entrance.** Ionic current blockades recorded through a single α-HL at an applied voltage ΔV = +130 mV in a 2 M KCl electrolyte buffered at pH=8, induced by freshly dissolved HBeAg* (60 nM) added on the *cis* chamber (a) and after two freeze-thaw cycles (b). The occurrence of blockade events is visibly decreased in the latter case, even at a higher concentration of the HBeAg* (90 nM). Herein, the symbol '*' associated with the HBeAg denotes that the purchased antigen (see Materials and Methods section).

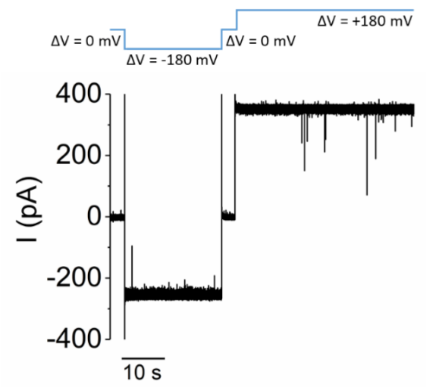

Figure S4. **The polarity of the transmembrane voltage critically sets the occurrence of monoclonal Ab(HBeAg)-α-HL nanopore interactions**. When present at bulk concentration of 100nM on the *cis* side of a lipid membrane isolating a single α-HL nanopore, collisional events reflecting nanopore-analyte interactions were seen only at *trans*-positive potentials.

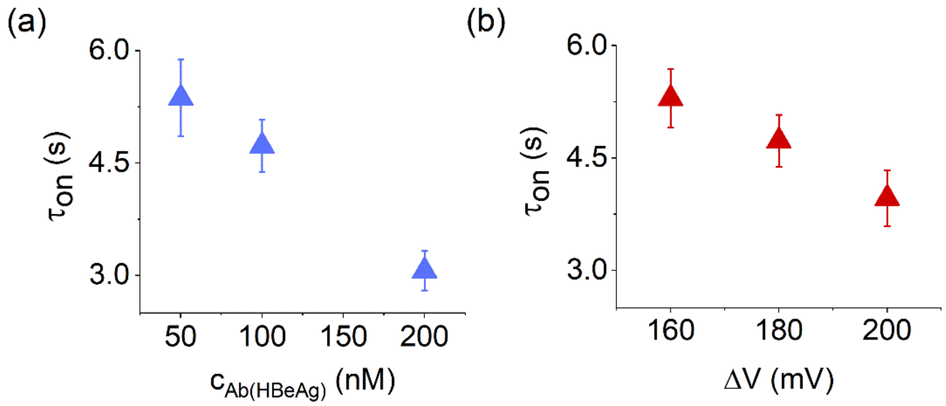

Figure S5. **The concentration- and voltage dependent association of the monoclonal Ab(HBeAg) antibody with a single α-HL nanopore.** (a) The average antibody-nanopore association time (τ_{on}) measured at increasing nanomolar concentration of antibody extracted from recordings made at an applied voltage ΔV = +180 mV. (b) The average antibody-nanopore

association time (τ_{on}) measured at increasingly applied voltages, with the Ab(HBeAg) added at a bulk concentration of 100 nM.

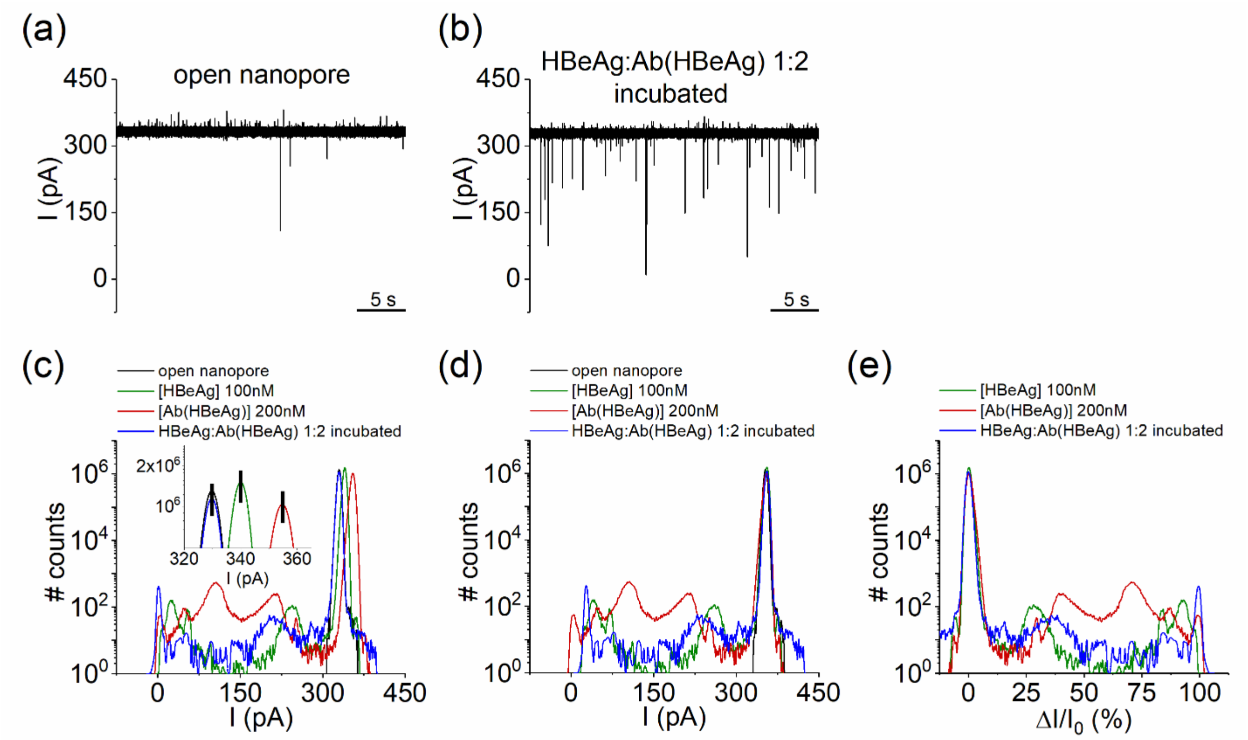

Figure S6. **Detection of pre-incubated HBeAg-Ab(HBeAg) complexes with the α-HL nanopore.** (a, b) Interaction between α-HL nanopore and a *cis* added mixture of HBeAg and Ab(HBeAg) (1:2 molar ratio), previously incubated for 2h 30min at room temperature. (c-e) The histograms showing the different conductive states of α-HL nanopore while interacting with the HBeAg-Ab(HBeAg) mixture (blue curve), relative to those measured with either HbeAg (green curve) or Ab(HBeAg) (red curve) alone. The applied voltage in such measuremernts was ΔV = +180 mV, and the recording buffer was similar as above.
